# My Early Relational Trust-Informed Learning (MERTIL) *for Parents*: A study protocol for a brief, universal, online, preventative parenting program to enhance relational health

**DOI:** 10.1371/journal.pone.0272101

**Published:** 2023-03-16

**Authors:** Jessica Opie, Leesa Hooker, Tanudja Gibson, Jennifer McIntosh

**Affiliations:** 1 School of Psychology & Public Health, La Trobe University, Melbourne, Australia; 2 Judith Lumley Centre, School of Nursing and Midwifery, La Trobe University, Melbourne, Australia; 3 La Trobe Rural Health School, La Trobe University, Bendigo, Australia; PLOS (Public Library of Science), UNITED KINGDOM

## Abstract

**Background:**

Early relational health is a key determinant of childhood development, while relational trauma in the parent-infant dyad can instigate a cascading pattern of infant risk. Fortunately, early relational trauma is detectable and modifiable. In 2018, Australian Maternal and Child Health (MCH) nurses participated in MERTIL (My Early Relational Trauma-Informed Learning), a program to identify and prevent relational trauma. Program evaluations revealed nurses felt competent and confident to identify and respond to relational trauma; however, response capacity was inhibited by inadequate parent referral options. In response, MERTIL *for Parents* (My Early Relational Trust-Informed Learning) was developed, which is an online, evidence-based, self-paced parenting program that focuses on enhancing parental knowledge of relational trust and its significance for infant development. This low-cost, accessible prevention resource targets emerging relational concerns to reduce later service system engagement. The potential for universal preventative online programs that target parental and relational wellbeing remains under-explored. This paper reports on a protocol for implementing a MERTIL *for Parents* pilot study describing practitioners’ and parents’ perspectives on program feasibility and efficacy.

**Methods:**

This study is a mixed methods, parallel armed, uncontrolled, repeated measures design. We aim to recruit 48 Australian MCH practitioners from the states of Victoria and New South Wales. These professionals will in turn recruit 480 parents with a child aged 0–5 years. All parents will receive MERTIL *for Parents*, which entails a 40-minute video, tipsheets, posters, and support resources. Parent data will be obtained at three periods: pre-program, program exit, and program follow-up. Practitioner data will be collected at two periods: pre-parent recruitment and program follow-up. Data collection will occur through surveys and focus groups. Primary parent outcomes will be socioemotional assessments of program efficacy. Practitioners and parents will each report on program feasibility.

**Discussion:**

This protocol describes the feasibility and efficacy of a new online parenting program, MERTIL *for Parents*, with pilot field studies commencing in March 2023. We anticipate that this resource will be a valuable addition to various child and family services, for use in individual support and group work.

## Study protocol introduction

Early relational health is a key determinant of childhood development, while relational trauma in the parent-infant dyad can instigate a cascading pattern of infant risk. Fortunately, early relational trauma is detectable and modifiable. In 2018, Australian Maternal and Child Health (MCH) nurses based in the state of Victoria participated in MERTIL *for Practitioners* (My Early Relational **Trauma**-Informed Learning), a program to identify and prevent early relational trauma (www.mertil.com.au). Program evaluations, as assessed via surveys and focus groups, revealed most MCH nurses felt competent and confident to identify and respond to relational trauma; however, response capacity was inhibited by inadequate parent referral options for those with sub-clinical presentations. In response, MERTIL *for Parents* (My Early Relational **Trust**-Informed Learning) was developed. MERTIL *for Parents* is an online, evidence-based, self-paced parenting program that focuses on enhancing parental knowledge of relational trust and its significance for infant development. This low-cost, accessible prevention resource targets emerging relational concerns to reduce later service system engagement. The potential for universal preventative online programs that target parental and relational wellbeing, as well as the development of early relational trust, remains under-explored.

This protocol describes the feasibility and efficacy of a new online parenting program, MERTIL *for Parents*, reporting on healthcare practitioners’ and parents’ perspectives on program feasibility and efficacy, with pilot field studies commencing in March 2023. This study is a mixed methods, parallel armed (i.e., practitioners and parents), uncontrolled, repeated measures design. We aim to recruit 48 MCH nurses and allied health practitioners who will in turn recruit 480 parents with a child aged 0–5 years (inclusive). All participants will receive access to the MERTIL *for Parents* program, which entails a 40-minute video, tipsheets, posters, and support resources. Parent and practitioner data collection will occur through surveys and focus groups. Primary parent outcomes will be socioemotional in nature to examine program efficacy; such outcomes will include child-parent relationship quality, parental reflective functioning, and child emotional functioning. Practitioners and parents will each report on study program feasibility. It is anticipated that this resource will be a valuable addition to a wide range of child and family services, for use in individual support and group work.

### Background

Children develop in a relational context with everyday parent-child interactional patterns contributing to their *relational health* [1]. Relational health in the infant-parent dyad is construed as parental sensitivity, trust, responsiveness, and security, which together form the cornerstone of socioemotional development. Alternatively, *relational trauma* presents ongoing and accruing disruption to the infant’s context of early care. Unsurprisingly, relational health and relational trauma impact upon an array of child developmental domains, instigating a cascading pattern of child resiliency or risk. Importantly, relational health elements, including attachment organisation and parental sensitivity [2–4] are modifiable, for better or worse. With this knowledge, promoting positive caregiving via psychoeducation and increased parenting capacity is vital, enabling parents to promote child development, thereby affording their child the best start in life. Early parenting programs have been developed as a result.

### Preventative parenting programs

Preventative parenting programs can be universal, selective, or indicated. Universal programs are those available to all parents; selective programs target parents displaying above average risk; and indicated programs target parents showing signs or symptoms of emerging disorder [5]. Research has shown universal programs are well suited to mental health promotion, offering significant preventative public health benefit [6].

#### Universal face-to-face parenting programs

Universal parenting skills-based programs have historically been presented through varied in-person modes, including face-to-face lectures, workshops, and groups. Program examples include CANparent [7] and *ACT Raising Safe Kids* [8]. Such programs have been shown to enhance both child and parent mental health among an array of domains such as parental self-efficacy, mental wellbeing, satisfaction, and child internalising and externalising presentations [e.g., 7–9].

Despite the availability and evidence supporting these in-person parenting programs, many families, particularly those experiencing numerous vulnerabilities, face multiple barriers in accessing these programs [10, 11]. For parents who live in regional centres and rural or remote locations, attending in-person programs, that are typically city-based, is an accessibility obstacle. Transportation access is also an issue for in-person parent service participation [12, 13]. Such programs are additionally cost prohibitive to many due to their high in-person contact hours with practitioners [14]. The inflexible structure of such programs and rigid delivery approaches can be perceived as stigmatising by the individuals these programs are intended to reach. Capped attendance due to physical room size, occupational health and safety concerns, and the limited number of facilitators running sessions provide further shortcomings. Parent logistical barriers, such as managing various daily work and lifestyle demands have resulted in low in-person parent program enrolment and poor program attendance for enrolees [e.g., 5, 15–17]. Compounding these limitations, the COVID-19 pandemic saw an abrupt stop to most in-person treatment programs due to lockdowns and quarantine, severing preventative program resources at a time of heightened psychological vulnerability for parents and children alike [18].

#### Universal online parenting programs

Online or app-based program delivery provides an avenue to address these limitations, provided intended users have adequate technology access, reliable internet connection, and are digitally literate. Online universal parenting programs can be either entirely or partially self-guided. In partially self-guided programs, clinician support is provided at varying frequency via online, phone, or in-person modalities [19]. Prior meta-analytic research on the efficacy of self-guided and partially self-guided online parenting programs has reported benefits at the parent, child, and dyadic level. Specifically, at the parent level, programs have been shown to enhance socioemotional outcomes such as positive parenting perceptions, behaviours, satisfaction, self-efficacy, and confidence, while lowering parental mental health problems (e.g., anxiety, anger, depression, and stress) and negative parenting practices [20–25]. At the child level, programs have been shown to decrease emotional and behavioural problems [21–23, 25], while at the relational level, they can reduce negative parent-child interactions [23], negative parental discipline strategies, and parental conflict [24].

While both self-guided and partially self-guided online parenting programs are efficacious, the formation and implementation of wholly self-guided, universal, digital parenting programs has recently gained momentum [e.g., 15, 26]. The rapid uptake of such programs is particularly due to the ease of administration, scalability, sustainability, low-cost, and program standardisation. Further rationale for the uptake of online program delivery is due to the ubiquitous nature of internet access, allowing widescale dissemination. For example, 99% of Australians and 91% of Australian households have internet access [27], making this a delivery method that can reach most of the population, thus making these programs highly accessible. The self-directed nature of online programs may further allow parents to flexibly complete these programs in the security of their homes, thereby overcoming possible judgement and stigma felt from participating in in-person programs. Such benefits may in turn reduce costs and barriers to program engagement and enhance program reach.

Due to their significant preventative potential, online parent resources may reduce the need for later intervention. This may occur by supporting and motivating parents with new knowledge and behavioural skills to independently enhance their parent-child relationship, in turn reducing later burden on service systems. Public health initiatives such as these may empower parents to alter relational trajectories early on, within a preventative approach that is parent-led and strengths-based. This may assist to disrupt emerging disadvantage before reaching pathological thresholds. Equitable access through online platforms has become more important, particularly in the face of the COVID-19 global crisis. Further, evidence shows that these programs promote help-seeking [28]. Finally, from a research perspective, fully online content allows for the collection and tracking of unique data on training progress, completion times, and premature program cessation. Such data can be linked to outcomes and inform program improvements.

### Knowledge gaps

While numerous clear benefits exist in relation to online, self-delivered, universal parenting programs, certain gaps remain. Currently, there are few brief self-help, psychoeducational program options (either in-person or online) that are suitable for parents at the broad community level, to raise awareness of their role in promoting early relational trust and preventing relational trauma. Second, most parenting resources in this domain exist in the context of in-depth indicated parenting interventions (e.g., Parent-Child Psychotherapy) [1]. While these indicated programs are essential for dyads in need of targeted treatment, there is a clear need for preventative education resources for parents in the wider community wishing to enhance their understanding of child socioemotional development and the parent-child relationship. This could act as an initial independent healthcare measure and reduce the need for later targeted interventions. Further, outcomes of online parenting programs tend to focus on parents and, to a lesser extent, child outcomes [21, 24]. Few studies have reported dyadic outcomes and even fewer have reported familial outcomes associated with online parent program participation [e.g., 23]. Finally, despite MCH nurses completing MERTIL *for Practitioners* and providing parenting support, they do not have a recommended suite of online parenting programs within their toolbox to assist clients [29]. MCH service practice guidelines focus on health promotion, child development, and to a lesser extent parent mental health; however, practitioners need evidence-based interventions to enhance dyadic relationships. This study aims to address these resource and knowledge gaps.

## Methods

### Study aims

This paper reports on a protocol for the pilot implementation of a new self-directed, universal, online parenting program, MERTIL *for Parents* (My Early Trust-Informed Learning), within the Maternal and Child Health (MCH) sector. The aims of this study are twofold:

1. Determine the feasibility of MERTIL *for Parents*:
  - For practitioners: Examine the programs acceptability and utility for enhancing parent understanding of early relational health; assess the practical dimension of implementing the program within a busy service; and explore population reach.
  - For parents: Examine program acceptability and utility.
2. Evaluate the short-term efficacy of MERTIL *for Parents* across selected parental caregiver knowledge, perceptions (e.g., parenting confidence and competence), and behavioural domains (e.g., professional help-seeking amenability).

### Intervention development

**Fig 1:**
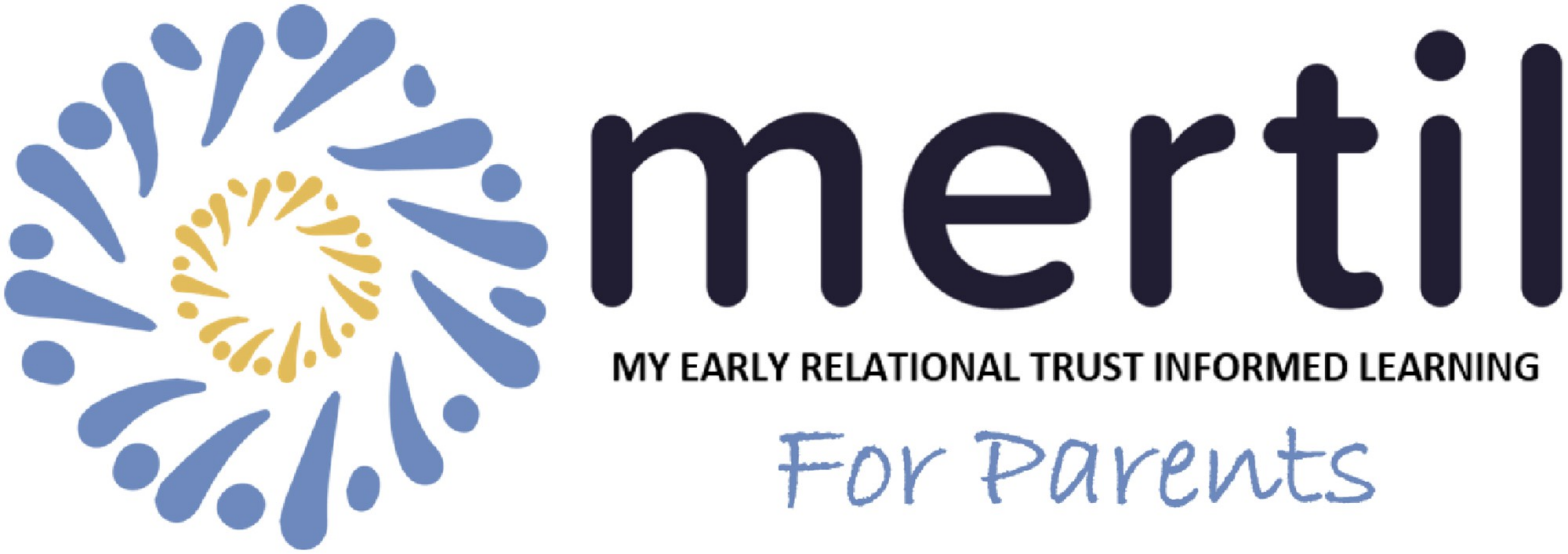
MERTIL *for Parents* logo.

MERTIL *for Parents* is a new universally available, self-directed, online psychoeducation resource for Australian parents focused on promoting parental knowledge and understanding of relational trust and its significance for infant development. This program is informed by evidence from attachment theory, developmental neuroscience, and psychoanalysis. As a preventative mental health resource, MERTIL *for Parents* aims to enhance infant-parent relational trust and relational awareness, in turn modifying parents’ attachment security-oriented beliefs, attitudes, and behaviours. MERTIL *for Parents* builds upon prior research by the authors. In 2018, over 1700 Australian MCH nurses from the state of Victoria participated in MERTIL *for Practitioners* (My Early Relational Trauma-Informed Learning), a 14-hour self-directed, interactive, professional development program for MCH nurses to identify and prevent relational trauma. The program evaluation from the 2018 Victorian cohort revealed that MERTIL *for Practitioners* enhanced competence and confidence in nurses’ identification of relational trauma [30]. However, nurse capacity to respond to such identified early relational trauma was inhibited by inadequate referral options for parents, particularly in rural and remote settings. More broadly, it was identified that the potential for universal online programs that target the development of early relational trust remains under-explored [30]. In response, MERTIL *for Parents* was developed. See Fig 1 for the MERTIL *for Parents* logo.

### The intervention

#### Intervention content

MERTIL *for Parents* is accessed via the internet and can be viewed on any online platform (i.e., phone, computer, or tablet). The primary component of MERTIL *for Parents* comprises a 40-minute pre-recorded video. Video content consists of four ‘chapters’, with each chapter approximately ten-minutes in duration. See Table 1 for a description of the MERTIL *for Parents* video content. Secondary program components include downloadable parent tipsheets, posters, and professional support contacts. All secondary program elements were developed by the study authors. See Table 2 for additional downloadable program resources.

**Table 1:** MERTIL *for Parents* video content.

Chapters	Content
**Chapter 1: Welcome to MERTIL *for Parents***	• Welcome & program background
	• Relational trust and its importance
	• Is this program right for me?
	• Selfcare while you listen
**Chapter 2: Why trust matters**	**Trust in parenting**
	• What does trust mean for a baby?
	• Parenting from a trust perspective
	• Infant-parent trust when the parent has relational trauma
	• Realistic child expectations: Dispelling unhelpful myths about babies
	• Practical suggestions to foster dyadic trust and shared delight
	• Realistic parenting expectations (the “good enough” parent)
	**Emotional capacities**
	Relational & child development knowledge:
	• Attachment relationships
	• Building strong trusting parent-child relationships
	• Promoting nurturing safe environments
	• The enduring impact of early relationships
	• Parenting capacities change with stress
	• Children’s stress tolerance & regulation capacities
**Chapter 3: Trust and trauma; rupture and repair**	**Building trust**
	Elements to building relational trust:
	• Parental predictability
	• Rupture and repair
	• Good stress, bad stress, and knowing the difference
	**Trust, trauma, and relationships**
	• Big relational ruptures
	• When trust has been tricky
	• When trauma is part of your parenting picture
	• Effects of parent trauma and violence on infant development
	• Family violence
	**The trust dance**
	• Practicing the parent’s steps
	• Understanding the child’s steps
**Chapter 4: Becoming your best parent, with support**	• Parent support and empowerment
	• Knowing when to seek professional help
	• Confidence in help seeking
	• Downloadable resources
	• Program summary

**Table 2:** Non-video MERTIL *for Parents* content.

Program element	Content
**Downloadable Tipsheets**	Tipsheet 1. Getting curious
	Tipsheet 2. All behaviour is a communication from your child
	Tipsheet 3. The good enough parent
	Tipsheet 4. Challenging behaviour
	Tipsheet 5. The importance of the early years
	Tipsheet 6. Putting your child’s experience into words
	Tipsheet 7. Being present in the moment, with your child
**Downloadable Posters**	Poster 1. All behaviour is communication
	Poster 2. The good enough parent
	Poster 3. Why trust matters
	Poster 4. Building trust and shared delight
	Poster 5. Does my baby need help to manage what they are feeling right now
	Poster 6. Rupture and repair
**Professional support**	Support sheet 1. International resources
	Support sheet 2. Australian state-specific resources

#### Program engagement

**Fig 2:**
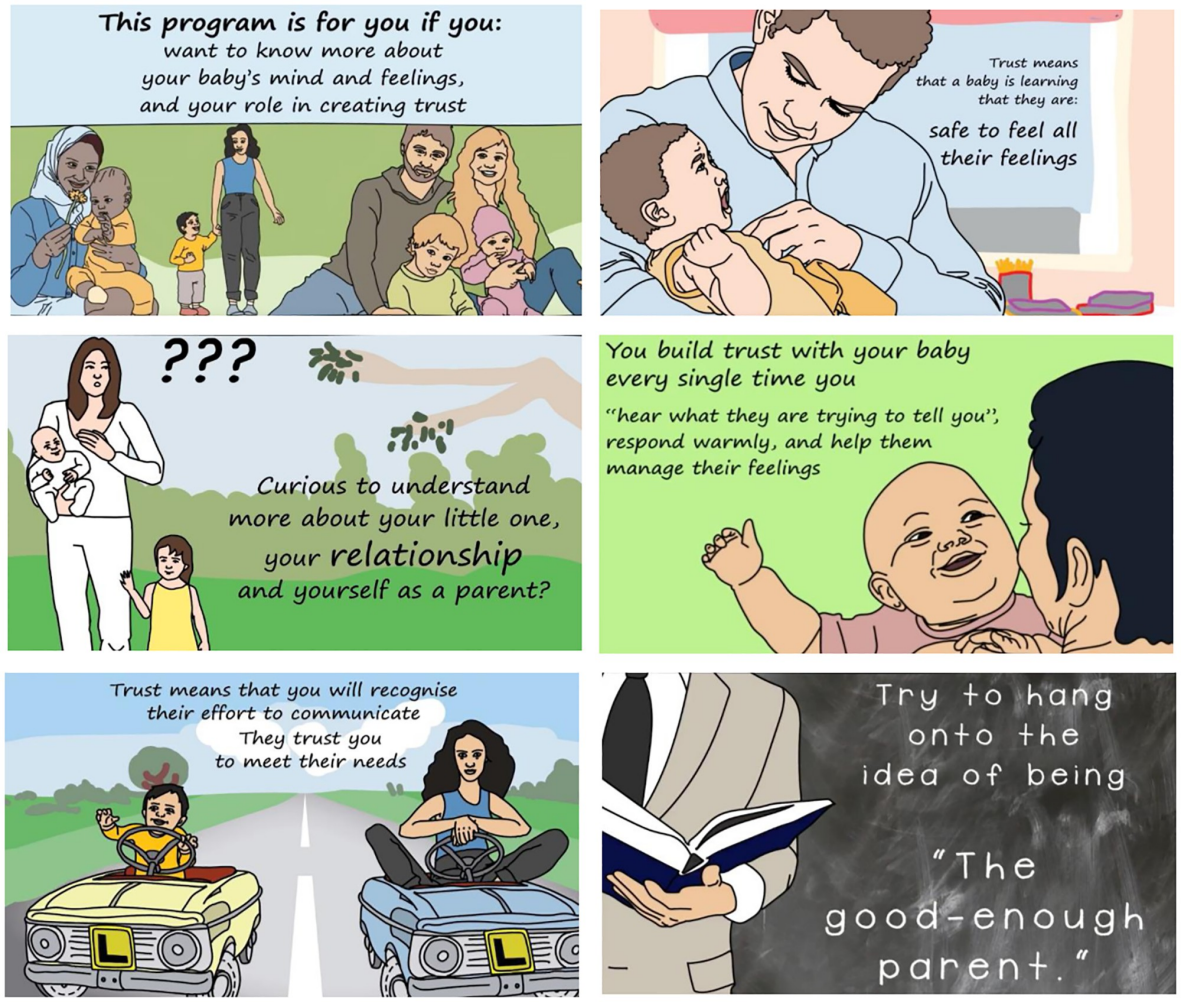
Screenshots from MERTIL *for Parents* video content.

Numerous steps were taken to ensure MERTIL *for Parents* is highly engaging, in order to i) enhance program enrolment, ii) deliver memorable content, and iii) maximise successful program completion. MERTIL *for Parents* was intentionally designed as a very-brief parenting program to counter the high dropout rates that have been observed in other online parenting programs [31]. The rationale for MERTIL *for Parents’* length was to introduce parents to the pivotal elements of relational trust, in a manageable, user-friendly, and time-efficient fashion that could be completed in one sitting or across several brief stints, as parents progress through the program at their own pace. A production team and animator translated the content into an engaging, animated format. See Fig 2 for screenshots of the MERTIL *for Parents* content.

*Program content & delivery*. To ensure the evidence-based narrative content was engaging and well received, content included:

1. Moments of humour to balance the program’s occasional heavy content.
2. Lay language to ensure the program’s content was successfully communicated to a varied parent audience.
3. An emphasis on safety, relatability, and acceptance; to this end, the narrator’s voice is warm, calm, non-blaming, and reassuring.

*Co-designed nature of* MERTIL *for Parents*. The MERTIL *for Parents* program content was co-designed and co-developed alongside parent consumers, early childhood mental health clinicians, and MCH nurses, from program commencement to completion. This collaborative process enabled the voice of ‘would be’ end-user parents and professional referrers to be heard; prior research has identified this co-design process as enhancing engagement, efficacy, and research applicability [32, 33]. Researchers and clinicians engaged in a multiphase feedback process, culminating in the resultant MERTIL *for Parents* program.

### Design

MERTIL *for Parents* will be examined via a parallel armed (i.e., practitioners and parents), uncontrolled, mixed methods, repeated measures study design. Practitioners will complete two assessments (pre-parent recruitment and three-month post-program) and parents will complete three assessments (pre-program, program exit, and three-month program follow-up). All practitioners and parent data collection and completion will be conducted independently and in parallel.

### Ethics statement

Ethical approval for this study was granted by the Human Research Ethics Committee at La Trobe University (HEC: 22096).

### Participants

**Fig 3:**
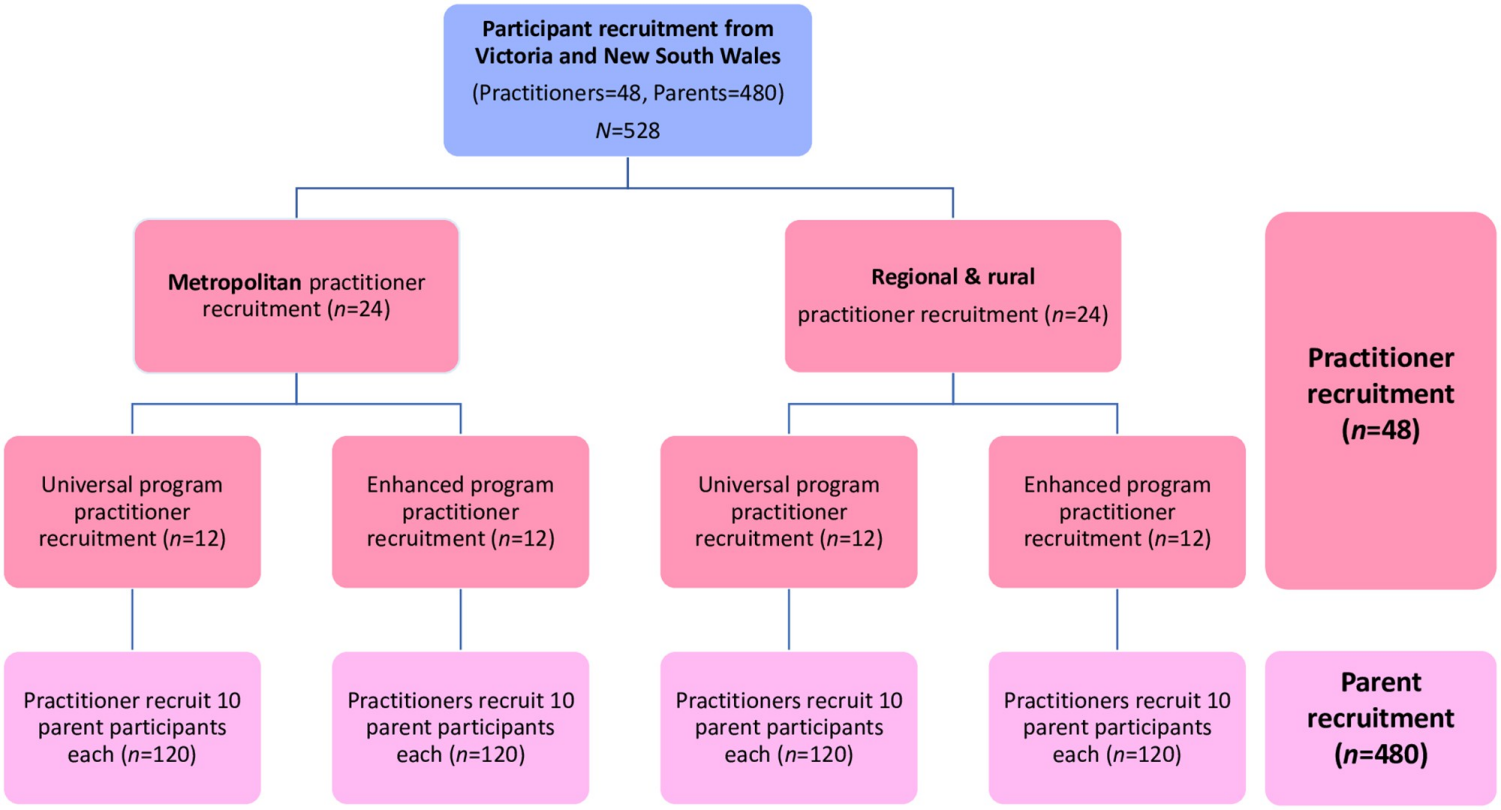
MERTIL *for Parents* recruitment diagram.

Eligible study participants will be twofold: i) Australian MCH nurses and perinatal and infant mental health practitioners, and ii) Australian parents. See Fig 3 for an outline of all study participants.

### Eligibility criteria

#### Practitioners

To be eligible for study participation, practitioners will: i) have completed MERTIL *for Practitioners*, ii) work at a participating Australian pilot site, in metropolitan or regional/rural Victorian and New South Wales, and iii) be able to make a follow-up contact (via a phone call, email, or face-to-face chat) to each of the parents they refer to the program, to gather feedback.

For practitioner participants, two key programs sit within the MCH umbrella: i) Universal services, and ii) Enhanced services [29]. Participants will be comprised of Universal and Enhanced service practitioners. Universal services are available to all families, while Enhanced services support ‘at-risk’ families. Universal practitioners will comprise of mostly MCH nurses from MCH centres, while Enhanced practitioners will include clinical staff from MCH centres *and* perinatal and infant mental health clinics. Enhanced service staff will include not only MCH nurses, but also psychologists, social workers, and psychotherapists. Study participation invitations will further be sent to practitioners who work in both the Universal *and* Enhanced services.

For practitioners who decide to participate, free access to MERTIL *for Practitioners* will be provided for the duration of the pilot study. All practitioners will have already completed this training; however, ongoing access will allow practitioners to refresh their knowledge of and skills in identifying early relational trauma, if necessary. Practitioners will also have ongoing access to MERTIL *for Parents*.

#### Parents

Consenting parent participants will: i) be the parent of a child 0–5 years (inclusive); ii) be recommended to participate in the program by an early childhood practitioner working at a participating pilot site; iii) aged 18 years and over; iv) currently reside in the state of Victoria or New South Wales, Australia; and v) feel comfortable completing questionnaires and program materials in English.

### Sample size/power calculation

Power analysis was conducted using the G*Power 3.1 software to determine the required sample size for this study [34]. A single group within study design would require 199 parent participants to identify a small to moderate statistically significant effect (.20) at *p* < .05, with 80% power. To account for parent participant attrition, we aim to recruit approximately 480 parents.

#### Sample

We aim to recruit 48 practitioners, consisting of 24 MCH practitioners from Universal MCH services, and 24 MCH practitioners from high-risk Enhanced services. These practitioners will in turn recruit the above-mentioned 480 parents.

### Recruitment of study populations

#### Practitioners

The authors will recruit through their existing MERTIL *for Practitioners* MCH nurse and perinatal mental health networks, ensuring a representative sample of MCH nurses and specialist perinatal and infant mental health clinicians from differing Australian geographic locations. Participating sites will include four Victorian and New South Wales metropolitan LGAs and four Victorian and New South Wales regional/rural LGAs, based on pre-existing profession networks. To further ensure representativeness, recruitment will include healthcare practitioners from the Universal and Enhanced MCH services, and from specialist perinatal and infant mental health clinics. All participating practitioners will provide online consent to their study participation.

#### Parents

Parents who meet inclusion criteria will be informed about the study by their MCH practitioner during a visit to a Universal MCH service (any of the scheduled visits from the initial post-birth home visit, consultations at 2, 4, & 8 weeks; 4, 8, 12, & 18 months; 2 & 3.5 years), or from any Enhanced MCH service engagement (i.e., MCH centre or specialist perinatal and infant mental health clinics). The practitioners will explain MERTIL *for Parents* and the associated research project, and if the parent verbally expresses interest, practitioners will direct them to the MERTIL *for Parents* website to register for the study and to access the materials (www.mertil.com.au). Practitioners will provide parents with a printed flyer with MERTIL *for Parents* details to aid this process. For parents who verbally agree to participate, practitioners will follow-up to gauge parents’ program responses.

During the study pilot phase, parents recommended to the program will go to the MERTIL *for Parents* website to join the research. Specifically, parents will be met with a webpage popup with a brief research overview and the option to hear more about the study. If parents select to hear more about the research, they will then be automatically prompted to enter their first name and email address. This will trigger access to an explanatory statement of the study and the option to consent to the research. Once the completed forms are submitted, parents will be invited to participate in a brief 10-minute pre-program baseline survey. Completing this survey will grant parents access to the MERTIL *for Parents* program.

### Procedure

#### Practitioners

Following informed consent, practitioners will attend a 120-minute web-based information and training session on MERTIL *for Parents*. This session will provide a study overview to promote program and pilot study familiarisation. Immediately following this session, practitioners will be asked to complete an online survey to report their first impressions of MERTIL *for Parents* regarding factors such as perceived program content, length, and suitability.

**Fig 4:**
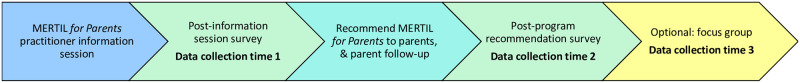
Flow diagram of MERTIL *for Parents* professional data collection timeline.

Practitioners will be asked to make contact with parents they refer to the program, within two weeks of recommending MERTIL *for Parents*. This follow-up contact will be via an informal face-to-face chat, phone call, or email. Once practitioners have recommended the program to parents, they will be invited to complete a second survey. This final practitioner survey will assess views on program usability, impactfulness, engagement, and ideas for program refinement and wider implementation. Online focus group interviews will follow to elaborate on survey responses, assess practitioner views and experiences of the program, potential barriers to implementation, and to identify further uses and applications of MERTIL *for Parents*. These elements will ensure MERTIL *for Parents’* co-designed nature endures. See Fig 4 for the professional data collection flow diagram.

#### Parents

During the study pilot phase, recommended parents who arrive at the MERTIL *for Parents* website will be welcomed to the pilot study. Recommended parents will be invited to read more about the study. If they consent, these parents will provide their contact details, agreeing to view the study’s explanatory statement and, if they wish, to complete the content form. Once complete, the pre-program parent survey will appear. Parents who arrive at the website, but who were not recommended to the program by their MCH practitioner, will be informed that the program is currently not widely accessible, but will be available to all following the current testing phase.

**Fig 5:**
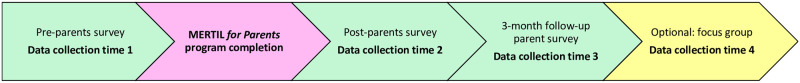
Flow diagram of MERTIL *for Parents* parent data collection timeline.

The brief parent surveys before, immediately after, and at 3-months follow-up comprise two sections: i) close-ended questions, and ii) open-ended questions. Upon completion of Survey 1 (10-minutes), participants can access the MERTIL *for Parents* program. Participants will have program access for 2 months from consent completion. Immediately upon MERTIL *for Parents* program completion, Survey 2 will be available (5-minutes). Three-months after completion of MERTIL *for Parents*, a follow-up survey (10-minutes) will be sent to explore sustained change. As needed, survey reminders will be sent via email at 7- and 14-days after the initial survey. If participants do not complete surveys after these communication bids, no further contact will be made. Consenting parents will participate in a qualitative interview, to further identify their program lived experience, as well as program strengths and weaknesses. See Fig 5 for an outline of parent data collection points.

During this research and piloting phase, MERTIL *for Parents* will be free of charge to recommended parents and caregivers who choose to participate in the research. Following this piloting phase and program refinement, MERTIL *for Parents* will be made broadly available.

### Measures

Our primary outcome will be to determine the efficacy of MERTIL *for Parents* in enhancing socioemotional development. Secondary outcomes include evaluating the feasibility of implementing MERTIL *for Parents* within Victorian and New South Wales MCH services.

#### Parents

*Baseline and three-month post-program questionnaires*. To evaluate program efficacy, participants will complete identical self-report measures pre-program and at three-months post-program (see Table 3); noting that demographic questions would have been completed pre-program and will not be repeated. Program efficacy questions will be both quantitative and qualitative in nature. Program efficacy will not be assessed at program exit as not enough time would have elapsed for meaningful parent change. All included measurement items were derived where possible from complete validated scales, single items from validated scales, and/or items from established Australian longitudinal cohort studies (i.e., Australian Temperament Project; Longitudinal Study of Australian Children); however, some study-generated items were also developed. Table 3 displays outcomes and associated measures that will be used to evaluate MERTIL *for Parents* pre- and post-program. For additional information, contact the first author.

**Table 3:** Pre- and post-MERTIL *for Parents* self-report outcomes and measures.

Outcome	Measure (reference)	Number of items
Study eligibility	Study-generated items	3
Demographics (including trauma items)	Study-generated items	19 (1 optional)
Parent-child relationship	Maternal Postnatal Attachment Scale [35]	4
	Perinatal Emotional Growth Index [36]	1
	Measures from longitudinal cohort studies [i.e., ATP; 37, LSAC; 38]	3
Child emotional functioning	Measures from longitudinal cohort studies [i.e., ATP; 37, LSAC; 38]	1
	Brief Infant-Toddler Social and Emotional Assessment [39]	5
Relational attunement	Parental Reflective Functioning Questionnaire [40]	5
Parenting experience & practices	Measures from longitudinal cohort studies [i.e., ATP; 37, LSAC; 38]	6
	The Parent Coping Scale [41]	1
Parent distress	Patient Health Questionnaire-4 [42]	2
Social support	Maternal Social Support Scale [43]	3
Professional help-seeking amenability	Study-generated item	1
Intimate partner safety	Family Law Detection of Overall Risk Screen [44]	2
Parent conflict	Measures from longitudinal cohort studies [i.e., ATP; 37, LSAC; 38]	2 (optional)
Qualitative questions	Study-generated items	2
**Total**		**60–63 (3 optional)**

*Note*. Light grey shading = Items not completed at the three-month follow-up survey; ATP = Australian Temperament Project; LSAC = Longitudinal Study of Australian Children.

In addition to the above items, in the pre-program and three-months post-program questionnaires, parents will be asked open-ended free-text response questions relating to the program. Pre-program questions will cover hopes for learning, and three-month post-program feedback questions will include items on program components that parents found most useful.

Embedded within this study is an internal program evaluation consisting of multiple-choice questions and open-ended text-response questions. Specifically, upon program exit (data collection time two) parents will be asked to complete a five-minute survey relating to program usability and program satisfaction. Example questions asked include: i) *How useful was the program for you*, *as a parent of a young child*? and ii) *What was most helpful about* MERTIL *for Parents*? This will be sent to parents via email with an attached secure link.

Parents’ online program usage data will also be automatically collected via the MERTIL *for Parents* website. This will record program engagement and completion elements such as i) program completion rates, ii) number of access occasions, iii) program components most/least accessed.

#### Practitioners

After practitioners hear from the parents they recommended MERTIL *for Parents* to, practitioners will be asked to complete a brief mixed methods online survey, allowing insight into practitioners’ perceptions of the program. Questions will include: i) *Would you recommend MERTIL for Parents to future parents*? ii) *Would you recommend colleagues encourage future parents to participate in MERTIL for Parents*? iii) *From your perspective*, *does MERTIL for Parents fill a current preventative health gap*? and iv) *Are there any existing gaps in the MERTIL for Parents program*? For each question response, options will be ‘yes’ or ‘no’, and then practitioner participants will be asked to expand upon their answer with free text.

### Focus groups

To further examine the feasibility and efficacy of MERTIL *for Parents*, four online Zoom-recorded focus groups will be held upon program completion. There will be two practitioner and two parent focus groups held. A subsample of volunteer practitioner and parent participants will join the optional focus groups with each group consisting of 4–6 parents or practitioners. A semi-structured interview schedule will guide focus group discussions. All focus groups will be audio recorded to allow for later transcription and analysis.

#### Practitioners

Example focus group questions will include: i) *Could you tell us about your MERTIL for Parents experience*? ii) *What was most relevant to you*? iii) *Can you see yourself in the future encouraging suitable parents to participate in MERTIL for Parents as a first intervention step*? and iv) *What are the long-term and short-term implications of programs such as MERTIL for Parents*?

#### Parent

Example focus group questions will include: i) *Could you tell us what it was like to participate in MERTIL for Parents*? ii) *Since completing MERTIL for Parents*, *what*, *if anything*, *has changed in your thinking*, *or feeling*, *or in your behaviour as a parent*? iii) *In MERTIL for Parents*, *what was most relevant to you*? and iv) *Would you recommend MERTIL for Parents to other parents*?

### Program data management

All professional and parent survey data will be disseminated through Moodle, an online learning management system stored on a secure server, with data transferred securely for analyses. All study data and participant information will be stored securely as per the criteria required by the La Trobe University Human Ethics Committee.

### Data analysis strategy

#### Quantitative

All statistical analyses will be conducted using the software IBM SPSS Statistics [45]. Descriptive statistics will be generated to identify professional and parent population characteristics and to evaluate parent and professional program perceptions. To determine parenting change in dyadic attachment, relational function, maternal mental health, from pre-program to three-months post-program, inferential statistics will be used (i.e., univariate, bivariate, and multivariate analyses) such as t-tests, chi-squared analyses, ANOVA, and regression analyses.

#### Qualitative

*Focus groups*. All parent and professional focus group interview data will be audio-recorded, transcribed verbatim, and deidentified. Thematic and content analysis will be used to identify themes and sub-themes from practitioners and parent transcript responses [46].

*Free-text surveys responses*. For practitioner and parent free-text survey responses, thematic analysis will also be used to identify response themes and sub-themes.

### Data collection commencement

Pilot field studies, including recruitment and data collection, will commence in March 2023 with data collection concluding in June 2023.

## Discussion

### Limitations and challenges

While this study is innovative, we foresee several program content and delivery method limitations: Firstly, it is possible that the brief self-guided content of MERTIL *for Parents* may not provide adequate content information and support (i.e., program dose too low). Second, as frontline services, practitioners may lack the time in session to explain and recommend parents to participate in MERTIL *for Parents*, due to competing demands and scheduling pressures. Third, as the study population pertains only to practitioners and parents located in Victoria and New South Wales, Australia, generalisability concerns will be present for interstate Australian and international populations. Fourth, as all MERTIL *for Parents* content is housed online, this may exclude those who lack the technology or skills to participate, especially as on-demand technical support will not be available for live participant trouble shooting. Further, because of this study’s non-randomised sampling strategy, cause-effect relationships cannot be drawn. That is, parents’ socioemotional outcome changes cannot be causally inferred by their MERTIL *for Parents* participation. Finally, as with all research, there is a risk that attrition rates may be high. All parent data will be self-report in nature, and interpretation of findings will therefore be subject to these limitations.

### Strengths and opportunities

There are multiple strengths to the present study: First, the mixed methods design of this study allows for greater understanding of the program’s impact compared with either qualitative- or quantitative-only designs. Second, diversity of parent demographics and needs will be ensured through recruitment of participants from both Universal and Enhanced MCH services, in both metropolitan and regional locations. Third, socioemotional-specific content may fill an important gap in the available online parenting program market. Fourth, the program’s reach is expected to be wide due to the online delivery method. Finally, strong completion is expected due to i) the program’s brief nature, ii) the engaging, non-judgemental, and supportive program content; and iii) the contribution of MCH nurses, infant mental health experts, and parents in co-designing the program.

### Conclusion

This study will examine implementation feasibility and efficacy of an evidence-based, online, universal, self-directed parenting program, MERTIL *for Parents*. Multi-informant data from parents and varied practitioners will inform future iterations of MERTIL *for Parents* and universal online relational programs.
